# Supplementary figures and images for: The SARS-CoV-2 Spike protein has a broad tropism for mammalian ACE2 proteins
Source: PLoS Biol. 2020 Dec 21;18(12):e3001016. doi: 10.1371/journal.pbio.3001016 (PMC7751883; doi:10.1371/journal.pbio.3001016)

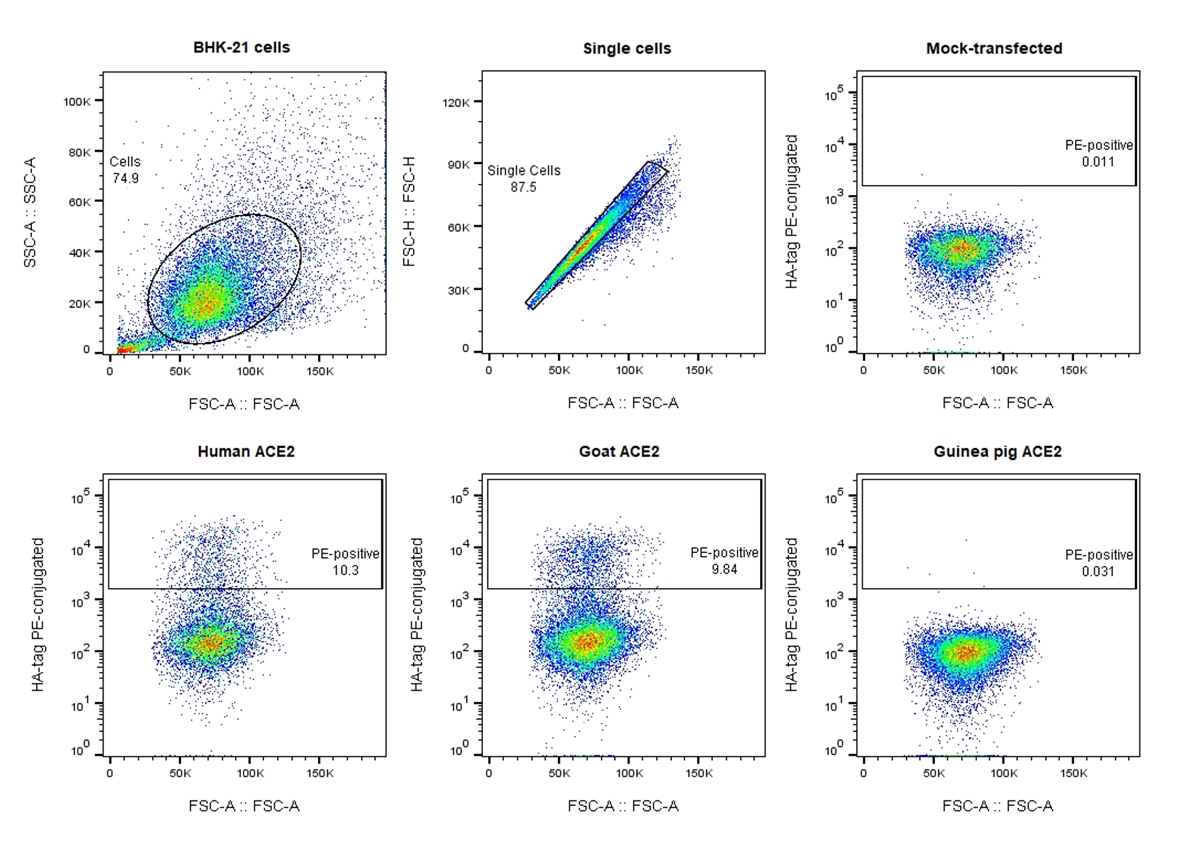

Supplement: S1 Fig — BHK-21 cells were transfected with a panel of species-specific ACE2-expressing constructs (see S2 Table). Cells were surface-stained with anti-HA PE-conjugated antibody. Live and singlet BHK-21 were gated as PE-positive, relative to mock-transfected cells (top panel). Representative datasets are shown for human, goat, and guinea pig ACE2 surface staining (bottom panel). ACE2, angiotensin-converting enzyme 2; BHK-21, baby hamster kidney; FSC-A, forward scatter area; HA, human influenza hemagglutinin tag; PE, Phycoerythrin. (TIF) [file pbio.3001016.s001.TIF]

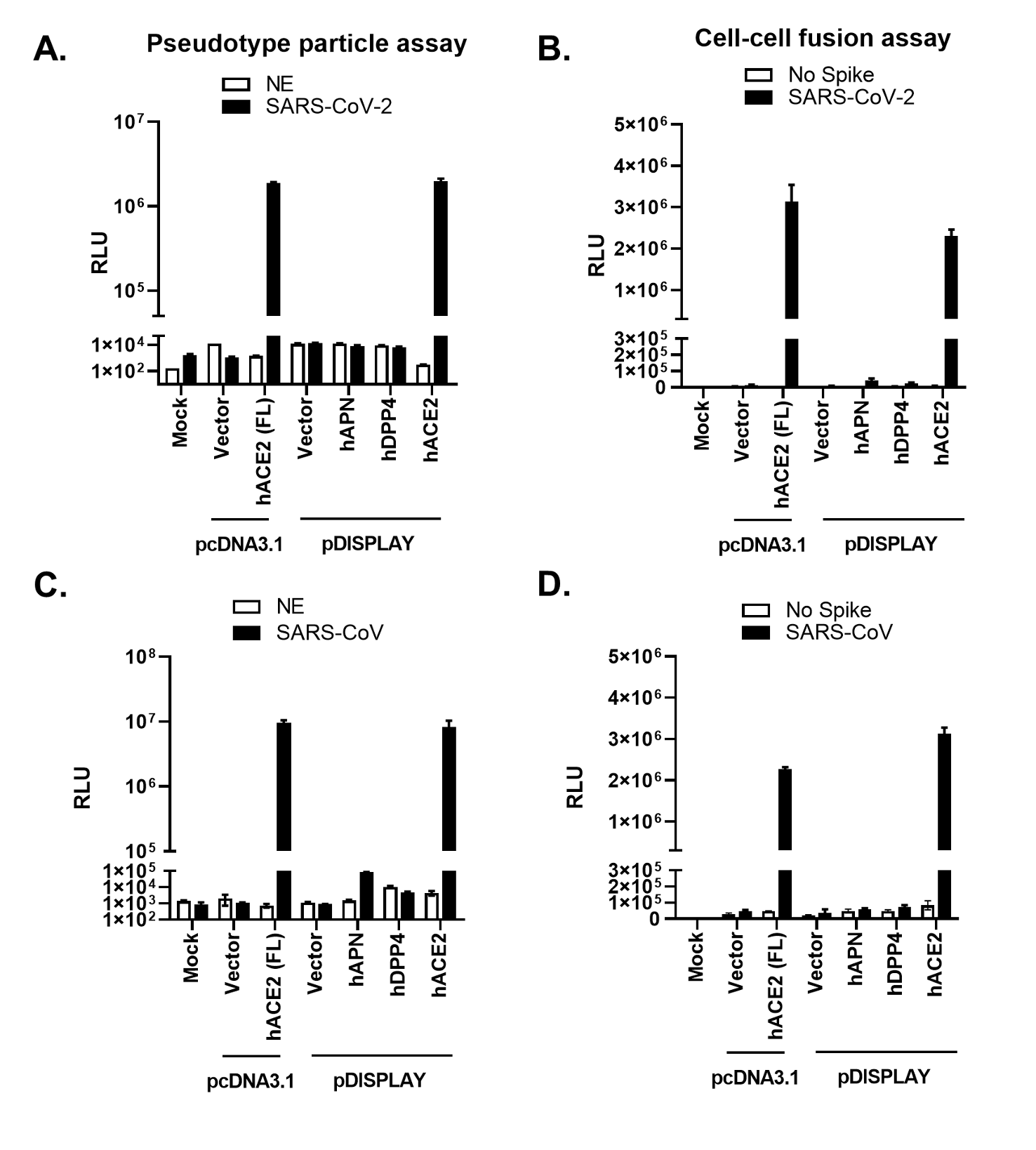

Supplement: S2 Fig — (A–D) Pseudotype and cell–cell fusion assays were established for SARS-CoV-2 (A and B) and SARS-CoV (C and D) using multiple internal controls. For the pseudotype assays NE lentiviral particles were generated, i.e., vector plasmid in place of a viral glycoprotein, to examine background levels of pseudoparticle entry. For the cell–cell fusion assay, mock-transfected effector cells were used (No Spike) to examine background levels of cell–cell fusion. In all subsequent experiments, “NE” and “No Spike” controls were compared against SARS-CoV-2 pseudoparticles or SARS-CoV-2 Spike-expressing effector cells (see S3 Fig). To validate our pDISPLAY approach, cells were transfected with expression constructs for full-length untagged hACE2 (FL) or a human ACE2 where the signal peptide was replaced with the murine Ig κ-chain leader sequence (hACE2) and the protein was tagged with the HA-epitope tag. In both instances, the corresponding vector controls, pcDNA3.1 and pDISPLAY, were separately transfected for comparison. The specificity of the SARS-CoV-2 and SARS-CoV assays were further confirmed by comparing hACE2-mediated fusion to human aminopeptidase N (hAPN) or dipeptidyl peptidase 4 (hDPP4) fusion, the coronavirus group I and MERS-CoV receptors, respectively. Lastly, in all assays, target cells representing un-transfected cells (mock) were also included. For pseudotype and cell–cell fusion assays, luciferase assays were performed in duplicate and triplicate, respectively, with the error bars denoting standard deviation. The data underlying this figure may be found in S1 Data. ACE2, angiotensin-converting enzyme 2; HA, human influenza hemagglutinin tag; hACE2, human ACE2; hAPN, human aminopeptidase N; hDPP4, human dipeptidyl peptidase 4; NE, non-enveloped; SARS-CoV, SARS Coronavirus; SARS-CoV-2, SARS Coronavirus 2. (TIF) [file pbio.3001016.s002.TIF]

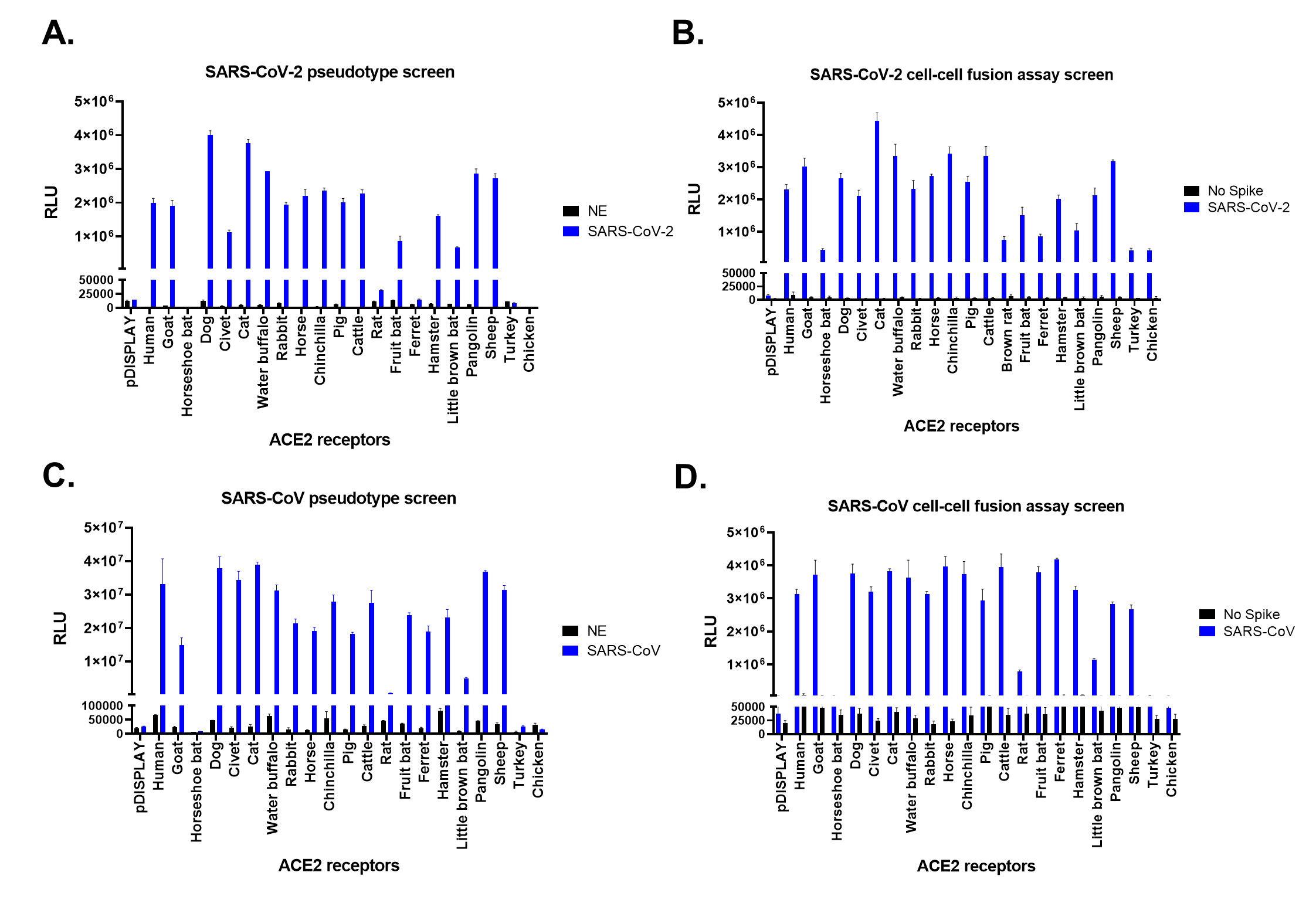

Supplement: S3 Fig — As per S2 Fig NE and No Spike controls were included in all assays, as well as a vector-only control (pDISPLAY). For pseudotype and cell–cell fusion assays, luciferase assays were performed in duplicate and triplicate, respectively, with the error bars denoting standard deviation. Representative data sets from individual experiments are shown; however, the heatmaps and XY correlative plots in Figs 2 and S5 summarise the results from 3 independent experiments performed on separate days. The data underlying this figure may be found in S1 Data. ACE2, angiotensin-converting enzyme 2; NE, non-enveloped; RLU, relative light units; SARS-CoV, SARS Coronavirus; SARS-CoV-2, SARS Coronavirus 2. (TIF) [file pbio.3001016.s003.TIF]

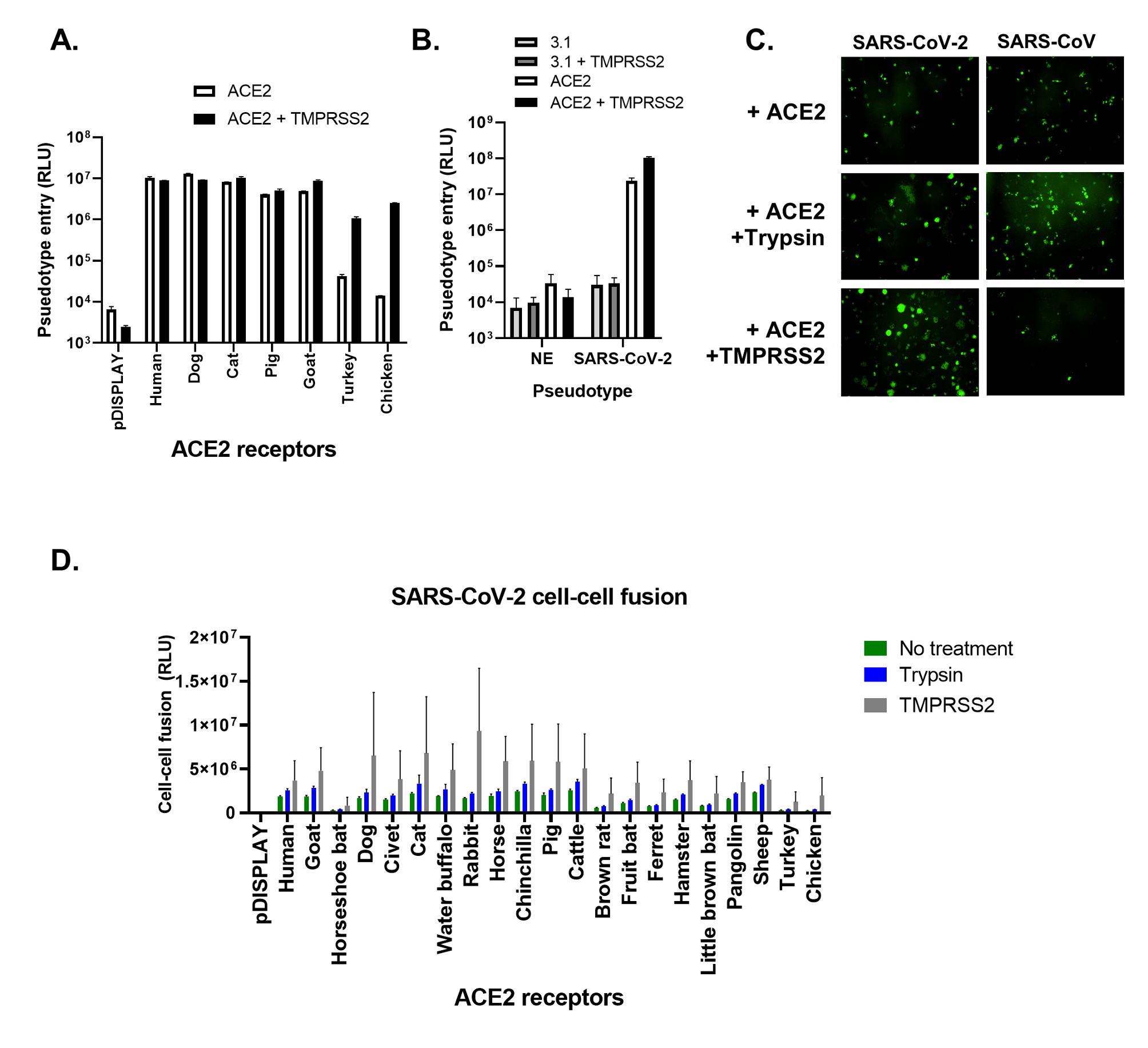

Supplement: S4 Fig — (A) Overexpression of human TMPRSS2 in target cells expressing various host ACE2 proteins has a negligible impact on SARS-CoV-2 pseudotype entry when a cognate ACE2 is present (human, dog, cat, pig, or goat); however, it disproportionately enhances entry with non-cognate ACE proteins (turkey and chicken), compared with ACE2 expression alone. Experiments were performed in biological triplicate and the mean RLU plotted, together with error bars denoting standard deviation. (B) Expression of TMPRSS2 alone does not support SARS-CoV-2 entry pseudotypes. NE HIV1 pseudotypes. (C) Trypsin treatment of effector cells or TMPRSS2 overexpression in the context of ACE2 leads to larger syncytia in SARS-CoV-2 cell–cell fusion assays. Trypsin treatment also increases SARS-CoV syncytium size, but TMPRSS2 expression observably reduces syncytia number. (D) Cell–cell fusion assay signals are higher when TMPRSS2 is co-expressed in target cells, including in target cells expressing non-cognate ACE2 proteins, e.g., turkey and chicken. Results represent the raw RLU signals from 3 independent experiments with the mean signal plotted and error bars denoting standard deviation. The data underlying this figure may be found in S1 Data. ACE2, angiotensin-converting enzyme 2; RLU, relative light units; SARS-CoV, SARS Coronavirus; SARS-CoV-2, SARS Coronavirus 2; TMPRSS2, transmembrane protease serine 2. (TIF) [file pbio.3001016.s004.TIF]

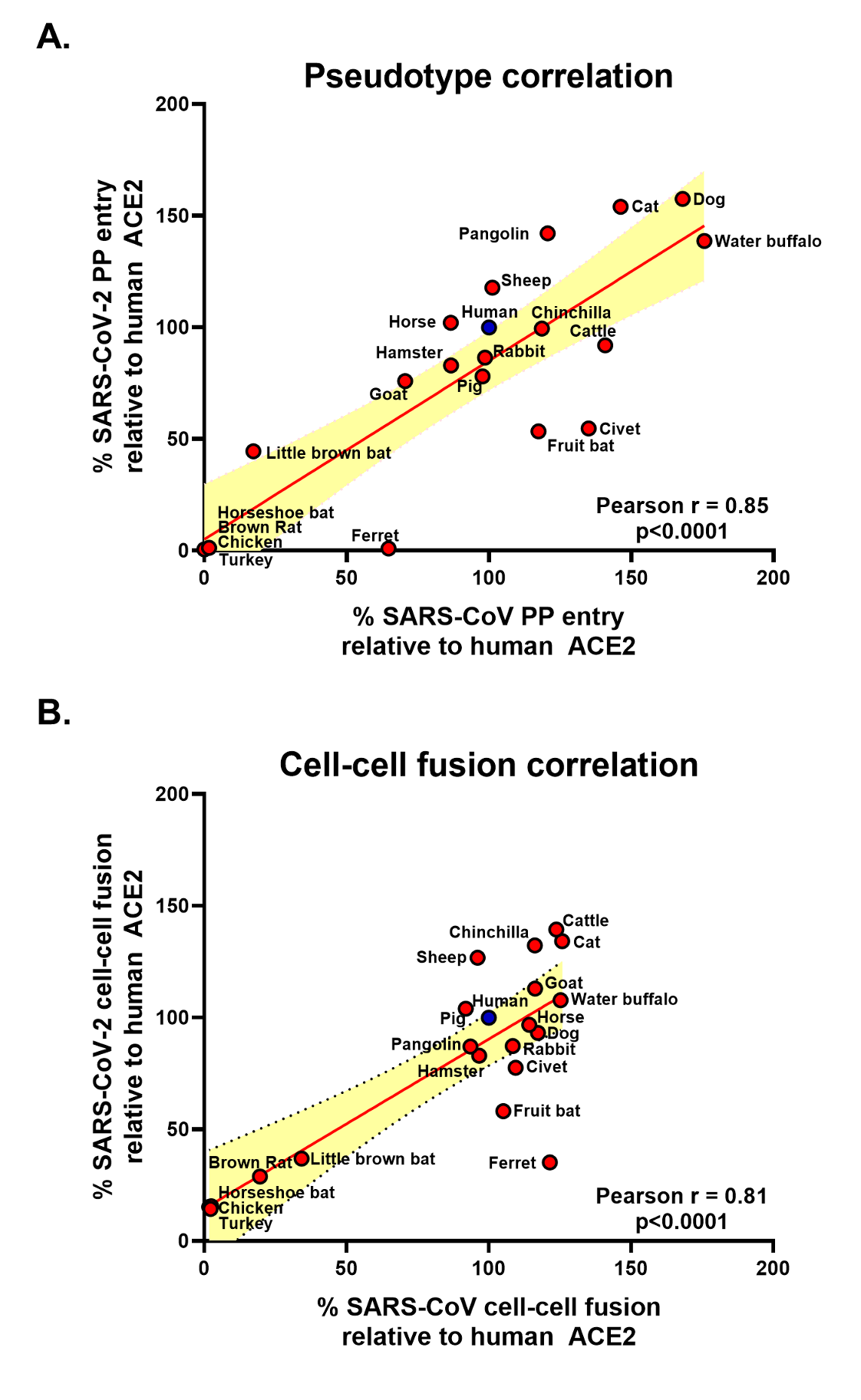

Supplement: S5 Fig — The receptor usage data for SARS-CoV-2 and SARS-CoV was examined by separately comparing the pseudotype (A) or cell–cell fusion (B) assay results on XY scatter plots. Each point represents the mean activity calculated from 3 independent experiments. The Pearson correlation was calculated, and a linear line of regression fitted together with 95% confidence intervals. All values are plotted relative to the entry or cell–cell fusion recorded for human ACE2 (blue circles). The data underlying this Figure may be found in S1 Data. ACE2, angiotensin-converting enzyme 2; SARS-CoV, SARS Coronavirus; SARS-CoV-2, SARS Coronavirus 2. (TIF) [file pbio.3001016.s005.TIF]

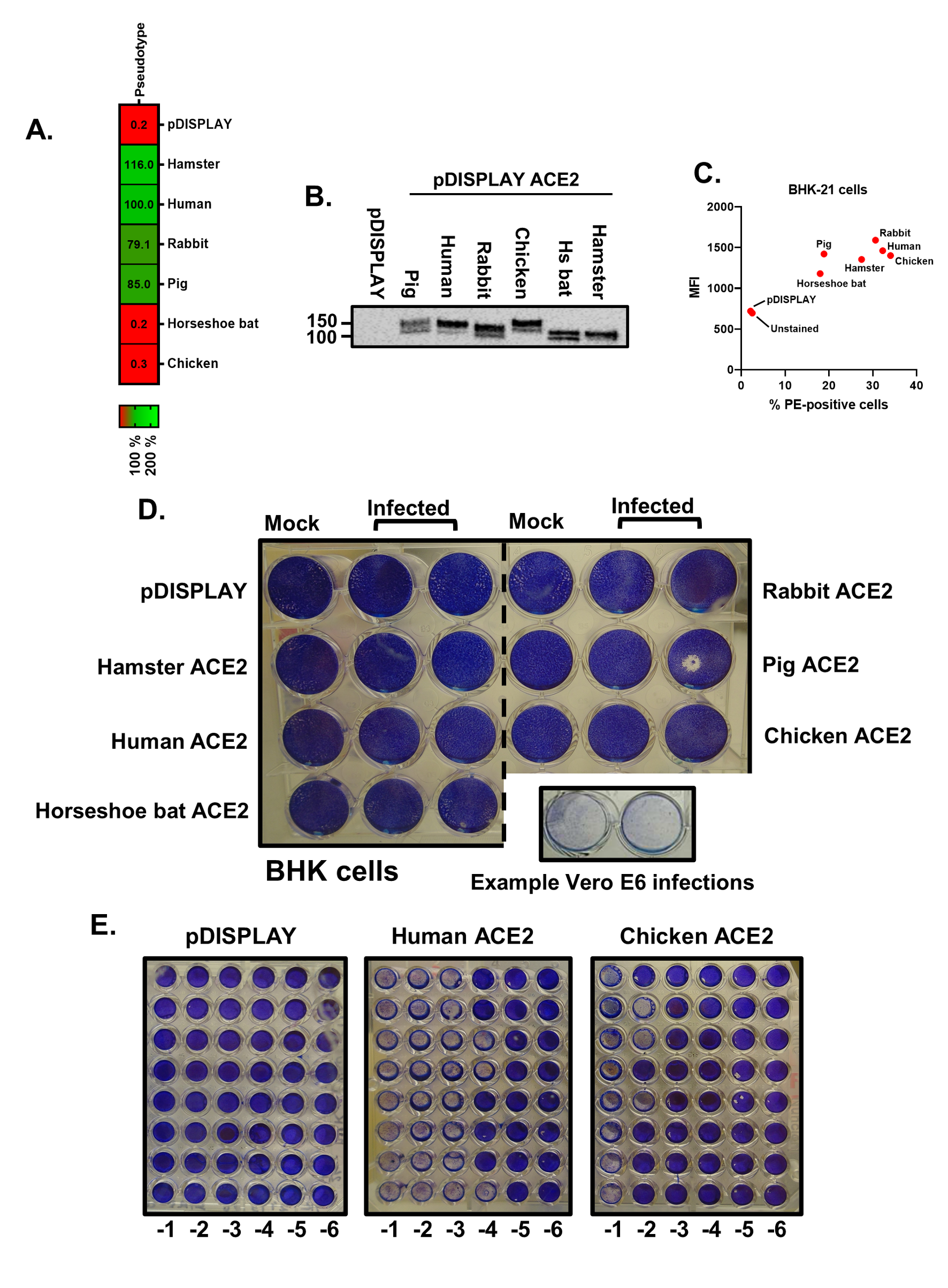

Supplement: S6 Fig — (A) SARS-CoV-2 pseudotype entry was assayed in BHK-21 transfected cells overexpressing ACE2 from the indicated species. Pseudotype infections were performed in triplicate, and the mean value was plotted on a heatmap following normalisation to human ACE2. Similarly, transfected target cells were lysed, and the ACE2 expression was analysed by western blot (B) or flow cytometry (C). (D) In parallel, BHK-21 cells were transfected with various ACE2-expressing constructs and infected with SARS-CoV-2 at an MOI of 1. Cells were fixed and stained at 48 hpi. (E) Prior to fixation, the supernatants from infected BHK-21 cells were removed for quantification of released virus by TCID-50. Representative images of these titrations, performed on Vero E6 cells, are shown (vector-only control [pDISPLAY] as well as human and chicken ACE2). The data underlying this figure may be found in S1 Data and S1 Raw Images. ACE2, angiotensin-converting enzyme 2; BHK-21, baby hamster kidney; MOI, multiplicity of infection; SARS-CoV-2, SARS Coronavirus 2; TCID-50, 50% tissue culture infective dose. (TIF) [file pbio.3001016.s006.TIF]

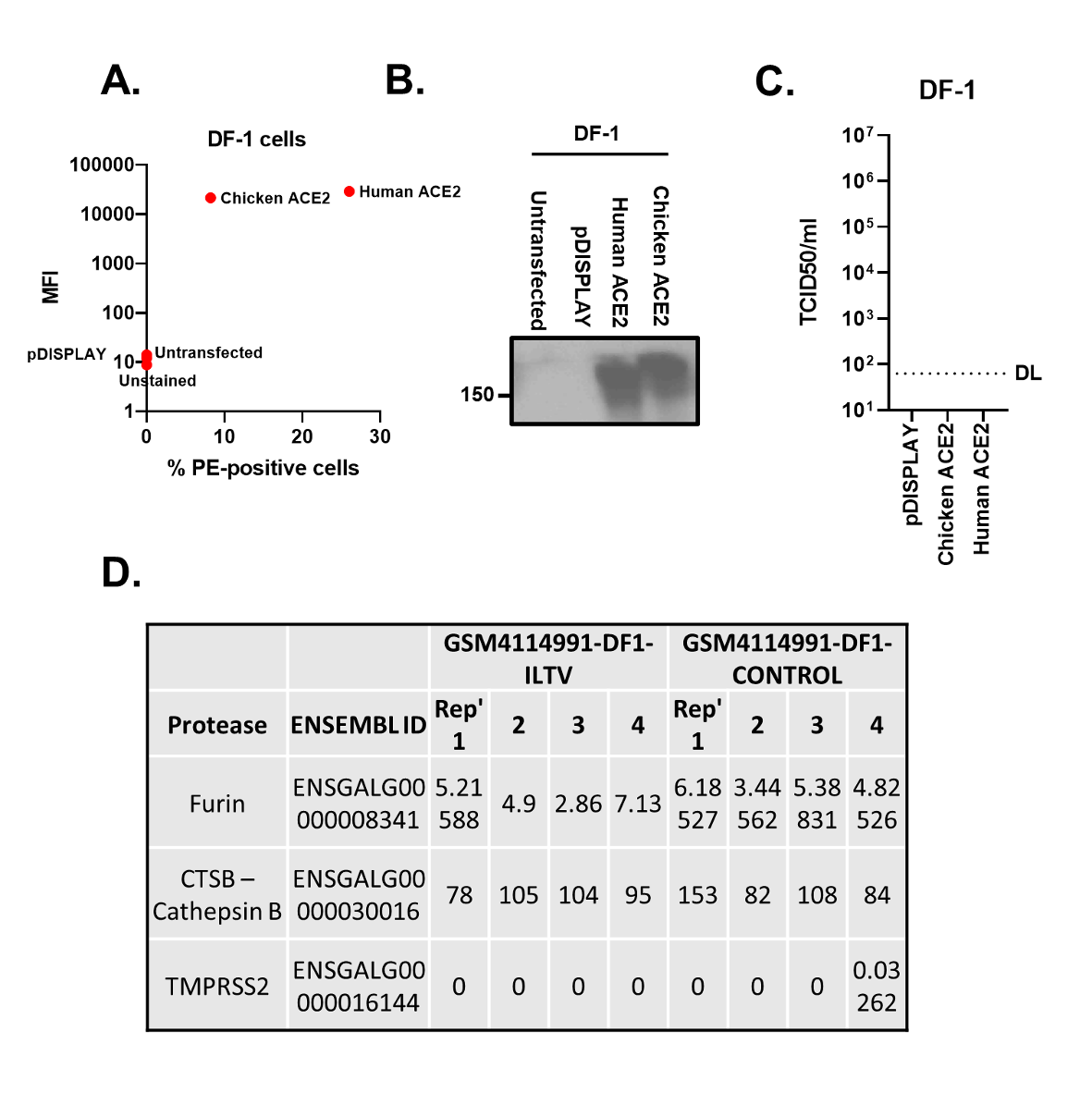

Supplement: S7 Fig — ACE2 expression in transfected DF-1 cells (as indicated) was analysed by flow cytometry (A) or western blot (B). For the flow cytometry, the MFI is shown for chicken and human ACE2-expressing cells (3264 and 9521 cells, respectively). For the controls, the total MFI of the HA labelling is shown as a small number of cells (<10) in the positive gate skewed the data. (C) Similarly, transfected DF-1 cells were infected with SARS-CoV-2 at an MOI of 1, in biological triplicate. Supernatant samples were harvested at 48 hpi for titration by TCID-50. The detection limit for the TCID-50 (DL) is indicated. (D) The mRNA expression levels of 3 proteases relevant for SARS-CoV-2 entry, Furin, Cathepsin B, and TMPRSS2, were evaluated in DF-1 cells by analysis of the publicly available gene expression database series GSE138648 ([18], dataset and methodology (GSM4114984) available at GEO; https://www.ncbi.nlm.nih.gov/geo/). Samples from both uninfected [Control] and ILTV DF-1 cells (4 biological replicates [Rep′] per condition) were examined and FPKM were calculated. The data underlying this figure may be found in S1 Data and S1 Raw Images. ACE2, angiotensin-converting enzyme 2; DF-1, chicken embryonic fibroblast cell line; DL, detection limit, FPKM, fragments per kilobase of exon per million reads mapped; HA, human influenza hemagglutinin tag; ILTV, infectious laryngotracheitis virus-infected; MFI, mean fluorescent intensity; MOI, multiplicity of infection; SARS-CoV-2, SARS Coronavirus 2; TCID-50, 50% tissue culture infective dose; TMPRSS2, transmembrane protease serine 2. (TIF) [file pbio.3001016.s007.TIF]

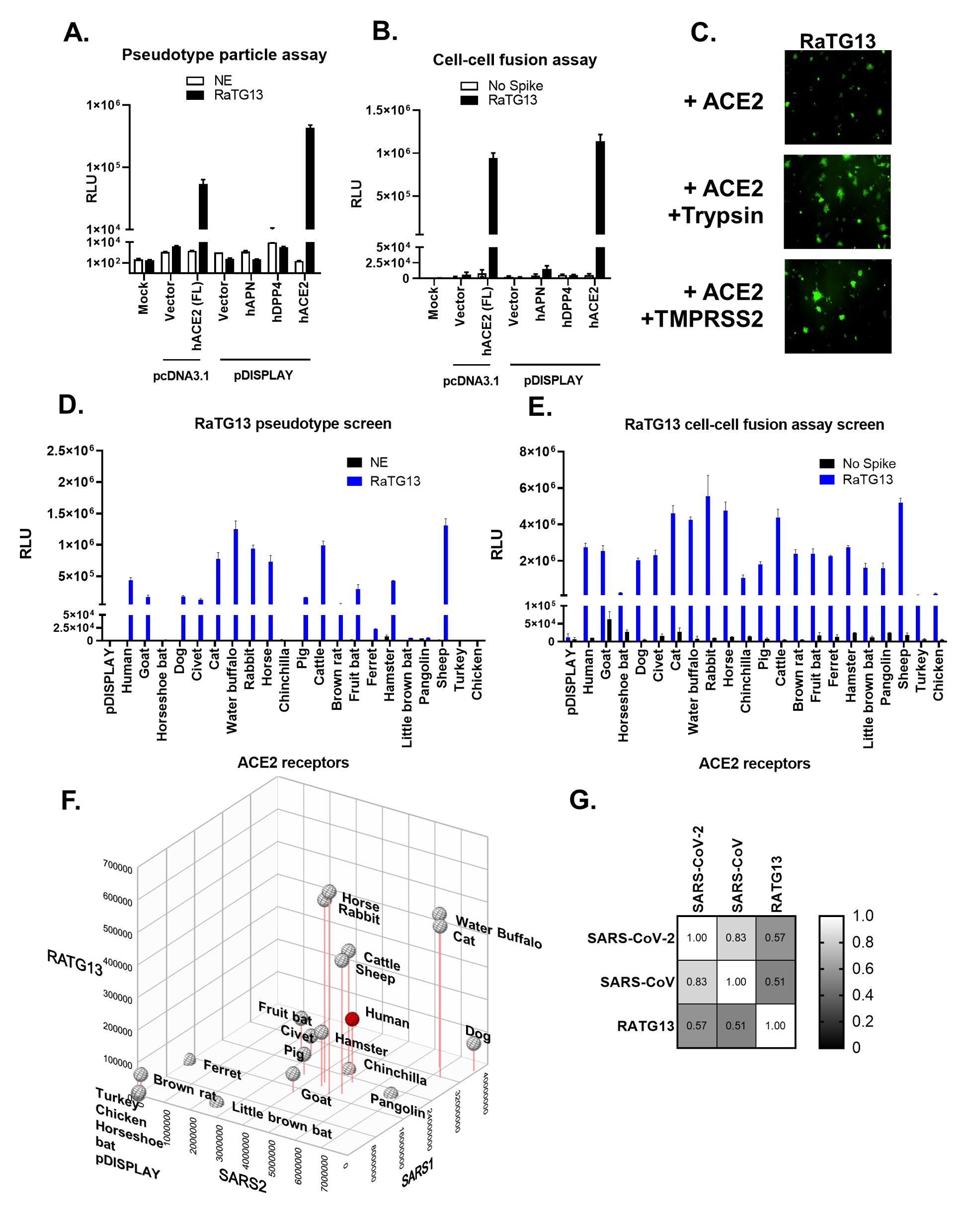

Supplement: S8 Fig — (A–C) Pseudotype (A) and cell–cell fusion assays (B and C) were established for RaTG13 as described for SARS-CoV-2 and SARS-CoV (see S2 Fig). (D and E) Receptor screening experiments were performed as described for SARS-CoV-2 and SARS-CoV (see S4 Fig). A representative data set is shown from a RaTG13 pseudotype and cell–cell fusion screen, with each screen eventually being performed in triplicate. (F) An XYZ scatter plot examining the receptor usage pattern of SARS-CoV-2 (SARS2; x), SARS-CoV (SARS1; z), and RaTG13 (y) Spike pseudotypes. The mean RLU data from 3 independent experiments is plotted with the human ACE2 value highlighted in red. (G) A Pearson correlation matrix for SARS-CoV-2, SARS-CoV, and RaTG13. The same raw RLU receptor usage values plotted in F were used for these calculations, with pDISPLAY values removed prior to calculation. The data underlying this figure may be found in S1 Data. ACE2, angiotensin-converting enzyme 2; RLU, relative light units; SARS-CoV, SARS Coronavirus; SARS-CoV-2, SARS Coronavirus 2. (TIF) [file pbio.3001016.s008.TIF]
