# Supplementary material for: The SARS-CoV-2 Spike protein has a broad tropism for mammalian ACE2 proteins
Source: PLoS Biol. 2020 Dec 21;18(12):e3001016. doi: 10.1371/journal.pbio.3001016 (PMC7751883; doi:10.1371/journal.pbio.3001016)
Supplement: S1 Table — (DOCX) [file pbio.3001016.s009.docx]

**S1 Table: Cell lines utilised in this study to quantify ACE2 mRNA levels and to assess virus permissibility.**

| **Cell line** | **Species** | **Organism** | **Cell type** | **Media and supplements** |
| --- | --- | --- | --- | --- |
| MDCK | Canine | *Canis familiaris* | Kidney, epithelial | EMEM |
| D17 | Canine | *Canis familiaris* | Lung, epithelial | EMEM |
| NBL-2 | Canine | *Canis familiaris* | Kidney, epithelial | EMEM |
| LLC-RK1 | Rabbit | *Oryctolagus cuniculus* | Kidney, epithelial | Medium 199: horse serum, 1.12 g/L sodium bicarbonate |
| RK-13 | Rabbit | *Oryctolagus cuniculus* | Kidney, epithelial | EMEM |
| SIRC | Rabbit | *Oryctolagus cuniculus* | Cornea, fibroblast | EMEM |
| BHK-21 | Hamster | *Mesocricetus auratus* | Kidney, fibroblast | EMEM |
| CHO | Hamster | *Cricetulus griseus* | Ovary, epithelial-like | Hams F-12K: 20 mM HEPES |
| DEDE | Hamster | *Cricetulus griseus* | Lung, fibroblast | McCoys 5a medium: 1.12 g/L sodium bicarbonate |
| NBL-6 | Horse | *Equus caballus* | Skin, fibroblast | EMEM |
| DF-1 | Chicken | *Gallus gallus* | Embryo, fibroblast | DMEM |
| LMH | Chicken | *Gallus gallus* | Liver, epithelial | Waymouth's medium |
| BT | Bovine | *Bos taurus* | Turbinate | DMEM |
| MDOK | Sheep | *Ovis aries* | Kidney, epithelial | EMEM |
| LLC-PK1 | Pig | *Sus scrofa* | Kidney, epithelial | Medium 199 |
| IPEC-J2 | Pig | *Sus scrofa* | Intestinal porcine enterocytes, epithelial | Hams F-12: 20 mM HEPES, 1% insulin/transferrin/ selenium (ITS) |
| PK15 | Pig | *Sus scrofa* | Kidney, epithelial | EMEM |
| ST | Pig | *Sus scrofa* | Testis, fibroblast | EMEM |
| COS7 | Monkey | *Cercopithecus aethiops* | Kidney, fibroblast | DMEM |
| Vero E6 | Monkey | *Cercopithecus aethiops* | Kidney, epithelial | DMEM |
| Marc 145 | Monkey | *Cercopithecus aethiops* | Kidney, epithelial | DMEM |
| McCoy | Mouse | *Mus musculus* | Fibroblast | EMEM |
| NIH3T3 | Mouse | *Mus musculus* | Embryo, fibroblast | DMEM |
| Duck embryo fibroblast | Duck | *Anas platyrhynchus domesticus* | Embryo, fibroblast | EMEM |
| QT35 | Quail | *Coturnix coturnix* | Muscle, fibroblast | EMEM |
