# Supplementary material for: The SARS-CoV-2 Spike protein has a broad tropism for mammalian ACE2 proteins
Source: PLoS Biol. 2020 Dec 21;18(12):e3001016. doi: 10.1371/journal.pbio.3001016 (PMC7751883; doi:10.1371/journal.pbio.3001016)
Supplement: S2 Table — (DOCX) [file pbio.3001016.s010.docx]

**S2 Table: Codon optimised ACE2-expression plasmids used in this study for receptor usage screens.**

| **Common name** | **Scientific name** | **Plasmid** | **Accession number** |
| --- | --- | --- | --- |
| Human (full length protein) | *Homo sapiens* | pcDNA3.1 | BAB40370.1 |
| Human | *Homo sapiens* | pDISPLAY | BAB40370.1 |
| Domestic cat | *Felis catus* | pDISPLAY | AAX59005.1 |
| Domestic dog | *Canis lupus familiaris* | pDISPLAY | ACT66277.1 |
| European rabbit | *Oryctolagus cuniculus* | pDISPLAY | XP002719891.1 |
| Horse | *Equus caballus* | pDISPLAY | XP001490241.1 |
| Guinea pig | *Cavia porcellus* | pDISPLAY | XM023562040.1 |
| Long-tailed chinchilla | *Chinchilla lanigera* | pDISPLAY | XM013506974.1 |
| Goat | *Capra hircus* | pDISPLAY | AHI85757.1 |
| Masked palm civet | *Paguma larvata* | pDISPLAY | AAX63775.1 |
| Water buffalo | Bubalus bubalis | pDISPLAY | XP006041602.1 |
| Least horseshoe bat | *Rhinolophus pusillus* | pDISPLAY | ADN93477.1 |
| Leschenault's rousette fruit bat | *Rousettus leschenaultii* | pDISPLAY | BAF50705.1 |
| Little brown bat | *Myotis lucifugus* | pDISPLAY | XP023609438.1 |
| Large Flying Fox bat | *Pteropus vampyrus* | pDISPLAY | XP011361275.1 |
| Pig | *Sus crofa* | pDISPLAY | NP001116542.1 |
| Cattle | *Bos taurus* | pDISPLAY | NP001019673.2 |
| Brown rat | *Rattus norvegicus* | pDISPLAY | NP001012006.1 |
| Domestic ferret | *Mustela putorius furo* | pDISPLAY | BAE53380.1 |
| Chinese hamster | *Cricetulus griseus* | pDISPLAY | XP027288607.1 |
| Malayan pangolin | *Manis javanica* | pDISPLAY | XP017505752.1 |
| Sheep | *Ovis aries* | pDISPLAY | XP011961657.1 |
| Chicken | *Gallus gallus* | pDISPLAY | QEQ50331.1 |
| Turkey | *Meleagris gallopavo* | pDISPLAY | XP019467554.1 |
