# Supplementary material for: The SARS-CoV-2 Spike protein has a broad tropism for mammalian ACE2 proteins
Source: PLoS Biol. 2020 Dec 21;18(12):e3001016. doi: 10.1371/journal.pbio.3001016 (PMC7751883; doi:10.1371/journal.pbio.3001016)
Supplement: S3 Table — (DOCX) [file pbio.3001016.s011.docx]

**S3 Table: β-coronavirus glycoproteins used in this study for receptor usage screens.**

| **Glycoprotein** | **Virus isolate** | **Backbone** | **Accession number** | **Source/**  **Reference** |
| --- | --- | --- | --- | --- |
| SARS-CoV-2 Spike | Wuhan-Hu-1 | pcDNA3.1+ with C-terminus FLAG tag | MN908947.3 | Synthesised from NCBI sequence |
| SARS-CoV Spike | ShanghaiQXC2 | pcDNA3.1+ with C-terminus FLAG tag | AAR86775.1 | Synthesised from NCBI sequence |
| Bat Coronavirus Spike | RaTG13 | pcDNA3.1+ with C-terminus FLAG tag | QHR63300.2 | Synthesised from Zhou,P et al., Nature 579, 2020. |
