# Supplementary material for: The SARS-CoV-2 Spike protein has a broad tropism for mammalian ACE2 proteins
Source: PLoS Biol. 2020 Dec 21;18(12):e3001016. doi: 10.1371/journal.pbio.3001016 (PMC7751883; doi:10.1371/journal.pbio.3001016)
Supplement: S4 Table — (DOCX) [file pbio.3001016.s012.docx]

**S4 Table: SARS-CoV-2 and RaTG13 glycoproteins with amino acid mutations in the RBD used in this study to generate chimeras.**

| **Glycoprotein** | **Amino acid mutations** | **Backbone** |
| --- | --- | --- |
| SARS-CoV-2 Spike | N439K  Y449F  E484T  F486L  Q493Y  Q498Y  N501D  Y505H  All, “chimera” | pcDNA3.1+ with C-terminus FLAG tag |
| RaTG13 Spike | K439N  F449Y  T484E  L486F  Y493Q  Y498Q  D501N  H505Y  All, “chimera” | pcDNA3.1+ with C-terminus FLAG tag |
