# Supplementary material for: The SARS-CoV-2 Spike protein has a broad tropism for mammalian ACE2 proteins
Source: PLoS Biol. 2020 Dec 21;18(12):e3001016. doi: 10.1371/journal.pbio.3001016 (PMC7751883; doi:10.1371/journal.pbio.3001016)
Supplement: S5 Table — (DOCX) [file pbio.3001016.s013.docx]

**S5 Table: qPCR primer sets used in this study to quantify ACE2 mRNA levels.**

| **Primer set** | **Target species** | **Target cell line** | **Forward primer (5’-3’)** | **Reverse primer (5’-3’)** |
| --- | --- | --- | --- | --- |
| **1** | Bird | DEF  QT-35  DF-1  LMH | TCCATGAAGCAGTAGGTGAAAT | CTCCACTTCTCCAGCATGTAAG |
| **2** | Rodent | BHK-21  CHO  DEDE  NIH3T3  McCoy | GTTAGAGAAGTGGAGGTGGATG | TGCAGGGTCACAGTATGTTT |
| **3** | Primate | Vero E6  COS7  Marc 145 | CCCTTTGGACAGAAACCAAAC | TTTCCCAGAATCCTTGAGTCAT |
|  | Rabbit | RK-13  SIRC |  |  |
| **4** | Pig | PK15  ST  IPEC | GAAGGGTGACTTCAGGATCAA | GCCATGTCATACTGGATGTG |
|  | Ruminant | MDOC  BT  NBL-6 |  |  |
|  | Dog | MDCK  D17  NBL-12 |  |  |
