# Supplementary material for: The SARS-CoV-2 Spike protein has a broad tropism for mammalian ACE2 proteins
Source: PLoS Biol. 2020 Dec 21;18(12):e3001016. doi: 10.1371/journal.pbio.3001016 (PMC7751883; doi:10.1371/journal.pbio.3001016)

# Figure 1F; Uncropped western blots

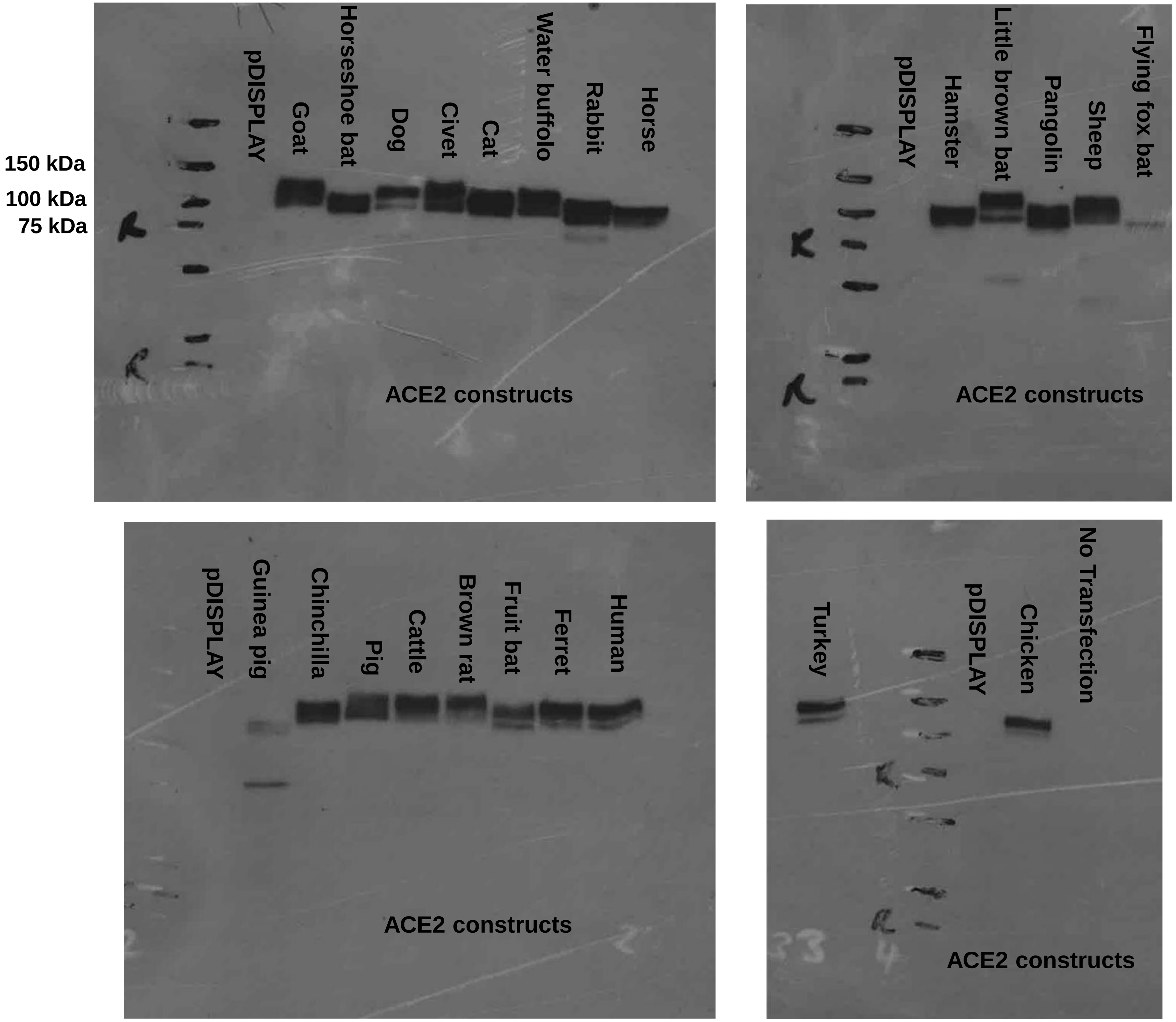

**Figure 4E; Uncropped western blots**

Anti-Flag blot (Spike)

Anti-GAPDH blot

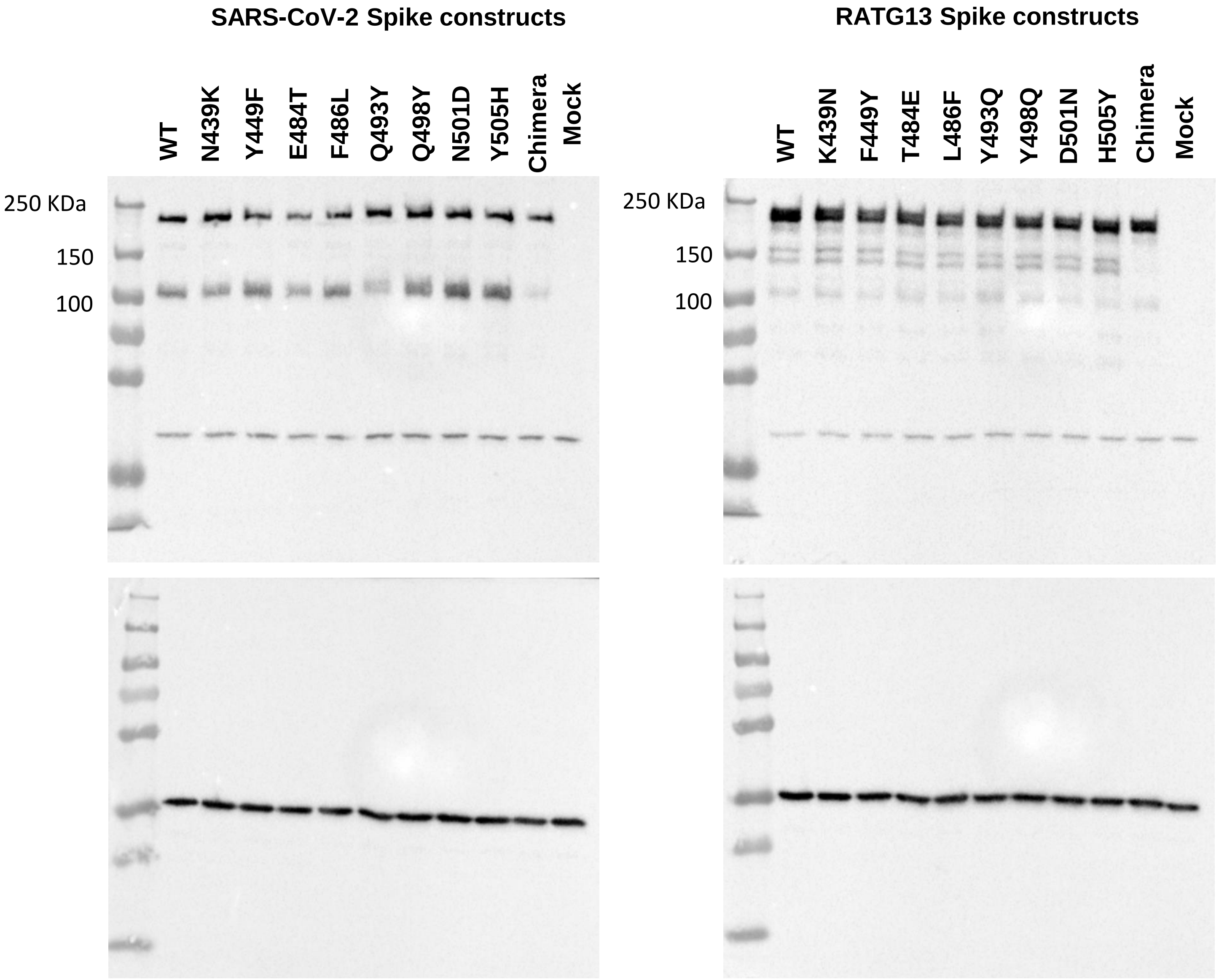

# Supplemental Figure 6B; Uncropped western blot

BHK + pDISPLAY
BHK pig ACE2
BHK human ACE2
BHK rabbit ACE2
BHK chicken ACE2
BHK horseshoe bat ACE2
BHK hamster ACE2

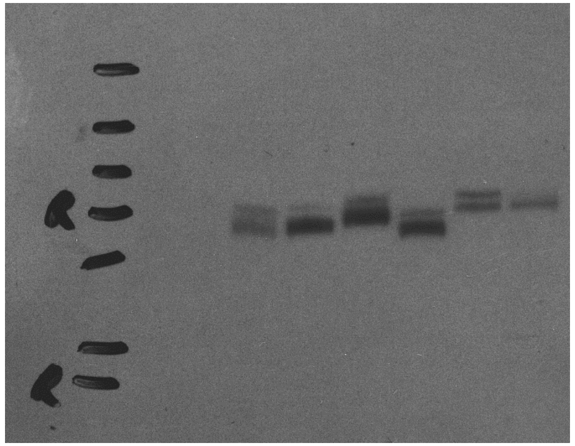

150 kDa
100 kDa
75 kDa

# Supplemental Figure 7B; Uncropped western blot

DF-1 untransfected
DF-1 pDISPLAY transfected
DF-1 human ACE2
DF-1 chicken ACE2

150 kDa
100 kDa

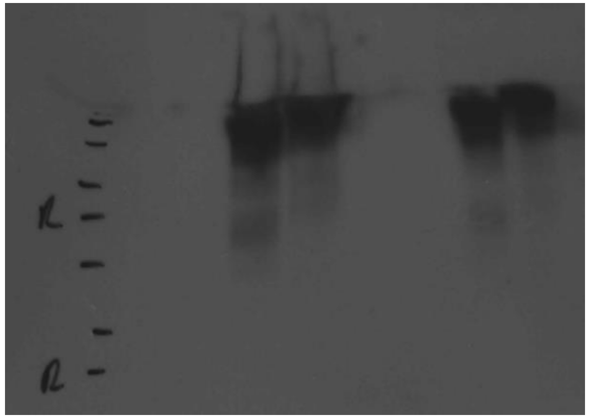

Supplement: S1 Raw Images — (PDF) [file pbio.3001016.s015.pdf]
