# Supplementary material for: The SARS-CoV-2 Spike protein has a broad tropism for mammalian ACE2 proteins
Source: PLoS Biol. 2020 Dec 21;18(12):e3001016. doi: 10.1371/journal.pbio.3001016 (PMC7751883; doi:10.1371/journal.pbio.3001016)
Supplement: Attachment — Submitted filename: Response to Reviewers.docx [file pbio.3001016.s016.docx]

**Dr. Dalan Bailey**

**The Pirbright Institute**

**Ash Road**

**Surrey, UK**

**28^th^ October 2020**

**Re: Response to Reviewers - PBIOLOGY-D-20-02408**

**‘The SARS-CoV-2 Spike protein has a broad tropism for mammalian ACE2 proteins’**

Dear PLOS Biology Editors and Reviewers,

Thank you for the detailed comments and suggestions regarding our submitted manuscript on SARS-CoV-2 host-range. We are happy that both reviewers realised the significant importance of the research and similarly appreciated the methodical nature in which it was performed. Before addressing the reviewer’s comments in detail, we would like to confirm that, together with this revision, we have submitted an Excel sheet containing our raw data and a PDF containing all uncropped Western blots. In addition, we have supplied a tracked changes version of the main text file, as requested. Lastly, we would like to thank PLOS Biology for supporting an anti-scooping policy. In relation to the Editor’s request and Reviewer 1’s comment that, *“This work has extensive overlap with recently published papers by Schlottau (Lancet), Zhao (JV), and Shi (Science) and even Zhou (Nature, initial characterization paper)”* we can confirm all of these papers are now cited and discussed in detail in the manuscript, in particular within a revised final paragraph of the discussion.

**Reviewer 1:**

**Minor Points:**

- **Amino acid 498 has also been changes in recent mouse adaptation work, it might be useful to comment on the flexibility/requirement for this contact residue in infection.** We thank the reviewer for this very useful suggestion/comment. The relevant paper from the Baric laboratory has now been discussed in detail in our paper. This study is particularly timely as it allows us to expand on why rat ACE2 is poorly utilised by SARS-CoV-2 Spike (Fig.2A). To this end we have changed the second paragraph of the discussion to acknowledge this paper’s findings, discuss the structural significance of residue 498, and to summarise how this effects our own interpretations of SARS-CoV-2 host-range in rodents (lines 343-370). Of note, we have also added reference to a new study describing the experimental challenge of deer mice that, like hamsters, appear susceptible to infection (lines 370-372).
- **Fig 5E - The chimera proteins don't appear to have cleavage products. Any reason or speculation as to why that would be?** Within the manuscript we state, “… the complete SARS-CoV-2 – RaTG13 chimeras containing all substitutions within the RBD (Fig.4C,D; Chimera) were non-functional and had altered electrophoretic mobility in western blots (Fig.4E) suggesting that the deleterious mutations (K439N or Y498Q) have a dominant effect in inhibiting ACE2 utilisation, perhaps by impeding correct folding of the mutated RaTG13 protein”. Whilst individual mutants at position 439 and 498 do not have the altered cleavage pattern highlighted by the reviewer, we think the cumulative effect of all eight mutations within the RBD combine to prevent correct folding and processing of Spike. The band at c. 100 KDa in the SARS-CoV-2 Westerns is likely the result of cleavage of the Spike’s polybasic site to S1 and S2 and it is interesting that this is lacking in the Chimera, perhaps providing further evidence of the incorrect folding of this mutant. To address this additional interpretation we have extended the sentence above to read, “…perhaps by impeding correct folding of the mutated RaTG13 and SARS-CoV-2 proteins, as well as potentially the cleavage of SARS-CoV-2 Spike into S1 and S2 (Fig.4E; left panel)” (lines 314-315).
- **The discussion is very long and detailed, the text associated with figures 5 and 6 would be better suited to the results section.** We acknowledge that the discussion is somewhat long; however, we feel this is the best place for our structural analysis as this reflects correlation of our own primary data with other published data sets. We feel it is important to separate primary data (in the results) from inferences based on structures published elsewhere. In addition although similar articles assessing the host-range of SARS-CoV-2 have now been published; we believe our structural interpretation of the data is more comprehensive and will serve as an important point of reference for future studies. However, to acknowledge the need for a more succinct discussion we have removed the technical comparison of the cell-cell and pseudotype assays, as this does not affect the overall interpretation of the data set. We have also refocused the final paragraphs (lines 480-515) to provide an improved comparison of our data to published studies on the in vivo pathogenesis of SARS-CoV-2 (experimental challenges or natural infections) – discussed below.

**Major Points:**

- **While some of these species have not been experimentally tested so far, at this point it is well demonstrated through naturally infections that many species are vulnerable to SARS2 infection - cats, dogs, tigers and mink have all been naturally infected. Hamsters have been used as a small animal model of infection in several publications. Previous work has shown that pigs and pig cells do not get infected while ferrets do get infected, how do the authors explain these discrepancies with their results?** We believe that for some animals the entry of SARS-CoV-2, i.e. through efficient ACE2 receptor usage, is the only significant barrier to infection (and potentially pathogenesis) - the most well characterised example being mice (now confirmed by Baric and other colleagues). This work, together with the recent evidence for SARS-CoV-2 infection of hamsters and deer mice (now referenced in the main text), provide a more detailed understanding of this restriction at the Spike-ACE2 interface. For other animals however, e.g. pigs where porcine ACE2 can be efficiently used, there clearly must be other restrictions – beyond entry – that play a role in determining pathogenesis. Determining the nature of these restrictions could have an important impact on our understanding of human disease - a good parallel being avian influenza, where mutations in the polymerase are required for enhanced replicative capacity in mammalian cells. To address this interesting dichotomy in more detail we have re-arranged the final paragraphs of the discussion and added more references to animal studies so that these points are clearer and collated together (lines 480-515).
- **Figure 3D - What proteases are present in the DF-1 cells? More detail is needed for this to be a convincing negative result.** We thank the reviewer for this interesting question. Acknowledging the need for a more detailed examination of the DF-1 data we analysed a published RNAseq dataset (Geo: GSE138648) of DF-1 cells to determine the mRNA expression levels of TMPRSS2, furin and cathepsin B proteases in this line (Sup.Fig.7D). In addition, in response to reviewer 2’s comments (see below), we repeated this experiment including the over-expression of human ACE2 as a positive control, and still did not see any evidence for DF-1 infection (Sup.Fig.7C). Based on these results we think it likely that restrictions to SARS-CoV-2 replication exist in DF-1s; however, the transcriptomic data indicates these blocks could theoretically be directly downstream of ACE2 binding, i.e. an absence of TMPRSS2 preventing cleavage of S2 to S2’. As discussed below (reviewer 2; comment 3), the absolute requirement for TMPRSS2 is still unclear, with functional redundancy in a cell-type specific manner a distinct possibility. However, given the published in vivo data demonstrating a lack of susceptibility in chickens and in ovo, we think it likely that post-entry blocks to replication in chicken cells also exist. Within the manuscript itself we have now modified the results and discussion sections to reflect this new experimental data and updated interpretations (lines 267-271 and lines 497-503).
- **What cell types from each of the species in Figure 3A were infected? Was there reason to believe these cells should be infectable (ie gastrointestinal or airway cells)? Similarly, DF-1 cells are chicken cells but what type of chicken cell, do they express the needed proteases?** The cell-type information for each cell line used in Fig.3A is provided in Supplemental Table 1; however, we admit this was not clearly indicated in the main text, which we have now addressed (line 233). ACE2 is broadly expressed in mammalian tissues, not just in the gastrointestinal tract and airway, so we tried to cast the net as wide as possible in designing these experiments. Indeed, a major cell line for SARS-CoV-2 propagation is Vero cells – a kidney cell. One of the early rationales behind this experiment was to provide a reference point for other researchers working on reverse zoonosis. Whilst a lot of this data is essentially negative (since ACE2 expression levels were low in many cells) we believe it is still important to show. However, we would be happy to move this to the Supplemental figures if the Editor thinks this is more appropriate. DF-1 cells are chicken embryo fibroblasts, which we have now specifically mentioned in the main text (line 264). Their protease expression profile is discussed above.

**Reviewer 2:**

**Comments:**

**1. In Fig1C, the ACE2 ectodomain map lists blue (divergent) to purple (conserved) residues. Clarification on what type of residue difference is considered divergent vs what type is considered conserved would be helpful to fully understand that figure.** Our analysis looked not just at per-residue similarity across species, but instead at the evolutionary rates at each position in the sequence as calculated using the ConSurf software. Sites that are evolving slowly (indicating purifying selection) are conserved, whereas those evolving more rapidly are divergent. We apologise that the method used for analysing sequence conservation was not clearly explained in the legend. We have now cited the paper describing this approach in the figure legend (line 749) and have provided more detail in the methods section as follows, “The selected sequences were aligned using MAFFT [51] and evolutionary conservation of amino acids was mapped onto the ACE2 structure using ConSurf [52], implementing a Bayesian framework to estimate the evolutionary rate of each amino acid in the sequence where slowly-evolving residues are conserved and fast-evolving residues are divergent” (lines 685-687).

**2. Fig2A. Heat map. It is unclear what the error or deviation from the mean is for the values listed here. Standard deviation or standard error need not be included in the heat map for every value but an error range could be listed in the legend (something like "standard deviation for the means range from 0.3-15%" for instance).** We apologise for this oversight and thank the reviewer for this useful suggestion. This information has now been added to the figure legend as requested (line 772-774).

**3. Sup Fig 6D. The chicken DF-1 cell line data is interesting. Overexpression of chicken ACE2 did not rectify lack of SARS-CoV-2 pseudotype entry. An interesting question results, is this due to some post-receptor binding factor or lack thereof? Why was human ACE2 not included in this expt to potentially address some aspects of this question? A further small concern with these expts is the low transfection efficiency (12%) for the DF-1 cells. Could this contribute to relative resistance to infection in DF-1 overexpression expt? Transfection efficiency for Pig ACE2 in BHK-21 cells was 20% and yielded robust virus entry so not sure if efficiency is an issue.** We thank the reviewer for this interesting observation and their suggestions. When compared to human ACE2, over-expression of chicken ACE2 in BHKs leads to approximately 1% of the nascent virus production following a high MOI infection (Fig.3C) – now emphasised in line 259. This data indicates that chicken ACE2 is bound by SARS-CoV-2, allowing some entry, albeit very poorly in comparison to cognate receptors (and close to the 0.5% value seen for pseudotypes; Fig. 2A). However, the reviewer is correct to postulate that this could also be because of a post-entry block, which our original dataset could not effectively delineate. As requested, we have now repeated this experiment including human ACE2 as a control. In this new experiment we did not see any evidence for nascent virus production in chicken or human ACE2 transfected DF-1s (Sup.Fig.7 and lines 267-271). Analysis of publicly available mRNA sequencing data of DF-1 cells identified that these cells lack TMPRSS2 expression (Sup.Fig.7D). However, the absolute requirement for this protein to facilitate SARS-CoV-2 entry is still not clear and there appears to be functional redundancy and alternative mechanisms for entry. In conclusion, it is likely that DF-1s provide a highly restrictive environment for SARS-CoV-2 replication, even in the presence of a cognate ACE2 (human), although this restriction may still be part of the entry pathway (a deficiency in TMPRSS2 cleavage of S2 to S2’), later in the life cycle, or indeed both. We have modified the relevant section of the results chapter for these experiments (lines 267-271), moving the related data into a new independent supplemental figure (Sup.Fig.7). We have also added a summary of the above comments into the discussion to address alternative interpretations of our dataset (lines 497-503). With regards to the transfection efficiency of the DF-1 cells: in three independent experiments we observed transfection efficiencies ranging from 8-17%. This is the main reason we used a higher MOI in these experiments (1), i.e. to ensure infection. Of note, the gates we applied for flow cytometry analsysis were stringent and these figures may represent a partial underestimation of ACE2-positive cells. In addition, as the reviewer rightly states a similar rate in pig ACE2-transfected BHKs yielded robust infection and of note in both cell types we saw efficient expression of ACE2 by Western blot (Sup.Fig.6 and Sup.Fig.7).

**Minor comments.**

**1. Sup Fig 6C. BHK-21 cell graph labeled as BHK-1?** This has now been changed as requested.

**2. Line 488. "syncytia formation" should be replaced by "syncytium formation". The latter term has historically been accepted and is most likely to be correct.** This text has been removed but we have checked the remainder of the manuscript to ensure the correct use of syncytium (singular) and syncytia (plural).

**3. When the Fig 1 guinea pig data is discussed in the results section, it would be helpful if there was a "(see Methods)" note or some other info to convey the details of the possible sequence issues on lines 702-4.** As requested, we have made this modification to the text (line 142)

Finally, we would like to thank the reviewers and editors once again for the time spent reviewing this manuscript. The bioarchived version of this manuscript is already receiving citations (PMID: 32935331 and 33101254) and we hope that the changes we have made to the main text, the additional experimentation we have performed, and the detailed explanations provided in this letter, can support acceptance and publication in PLOS Biology.

Yours sincerely,

Dalan Bailey

(on behalf of all other co-authors).
